# Evaluation of Radiological and Anatomical Features of Cervical Vertebrae in Adult Persian Cat

**DOI:** 10.1002/vms3.70109

**Published:** 2024-11-09

**Authors:** Peghah Derakhshi, Siamak Alizadeh, Mohammadreza Hosseinchi

**Affiliations:** ^1^ Faculty of Veterinary Medicine, Urmia Branch, Islamic Azad University Urmia Iran; ^2^ Department of Clinical Sciences Faculty of Veterinary Medicine, Naghadeh Branch, Islamic Azad University Naghadeh Iran; ^3^ Department of Basic Sciences Faculty of Veterinary Medicine, Urmia Branch, Islamic Azad University Urmia Iran

**Keywords:** anatomy, cervical vertebrae, persian cat, radiology

## Abstract

**Background:**

Radiographic studies of Persian cat's cervical vertebrae can provide us with valuable information used in identifying its anatomical features, investigating various species of Persian cats and evaluating their cervical pathologies.

**Objectives:**

The present study aimed to investigate the anatomical and radiological features of cervical vertebrae in Persian cats to create a comprehensive and accurate reference for the normal range of cervical bones and joints that can be used for clinical decision‐making and interpretation of radiographic findings in these cats.

**Methods:**

The present descriptive‐cross‐sectional study included 10 adult Persian cats, including 5 males and 5 females. All cats underwent radiography in the dorsoventral, ventrodorsal, left lateral and right lateral recumbency. Then, anatomical investigations were performed.

**Results:**

According to our findings, Persian cats were different from other cat species in some cervical vertebral characteristics, which can be suggested as comparative anatomy in these species. The most important differences were as follows: the C3 and C7 had the lowest and highest spinous process height (SPH), respectively. The atlas had the highest transverse process width (TPW), which was significantly different with other cervical vertebrae (*p* ≤ 0.05). Moreover, the cervical vertebrae were not significantly different in vertical diameter of cranial vertebral foramen (VDCrVF) and transverse diameter of cranial vertebral foramen (TDCrVF) (*p* ≤ 0.05). The caudal surface of the atlantic vertebral arch had two dorsal and ventral tubercles. Also, the ventral crest of the axis was quite indistinct in the cranial part while becoming prominent in the caudal part. The C3 spinous process was a wide, small tubercle, while the C7 spinous process was extended vertically. Finally, the C6 transverse processes had a large ventrocranial tubercle forming a sagittal plate with cranial and caudal parts separated by a notch.

**Conclusion:**

The accurate and comprehensive standard ranges obtained from the present study can be used for the interpretation of imaging results, clinical decision‐making, and finding the normal and abnormal sizes of the cervical vertebrae and their processes in Persian cats.

## Graphical abstract

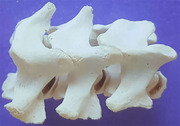

## 1 Introduction

Radiographic studies of Persian cat's cervical vertebrae can provide us with valuable information used in identifying its anatomical features, investigating various species of Persian cats, and evaluating their cervical pathologies. As one of the most famous feline breeds globally, the Persian or Iranian cat is a longhair breed with a round, flat face and a short muzzle. Its life span is about 10 years, which may reach up to 19 years under desirable conditions (Schmidt et al. 2022). These cats have blue, orange or bicoloured eyes, and the albino cats are often blue‐eyed (Wilhelmy et al. 2016). The current Persian cats, particularly the flat‐faced breeds, have brachycephalic syndrome, which is associated with a round, big skull and short nose.

On the other hand, the spine is an essential part of the feline skeleton that can be injured due to various causes, while such injuries can be diagnosed using different imaging modalities. However, interpretation of imaging findings is not possible without comprehensive information on the anatomical details (morphology and morphometry) of vertebrae in different feline breeds. As a non‐invasive diagnostic imaging modality, X‐ray radiography can provide us with precise and comprehensive details of the spine and vertebrae. It is obvious that sufficient knowledge of the normal details of each vertebra is essential for accurate diagnosis of vertebral abnormalities.

The feline spine, which can be evaluated using radiography, consists of vertebrae, intervertebral discs, meninges and spinal cord (De Decker, Warner, and Volk 2017). Bone abnormalities can be diagnosed using plain radiography (Bibbiani et al. 2022), while soft tissue problems can be detected using contrast radiography. Moreover, 3‐dimensional cross‐sectional imaging is highly beneficial for the diagnosis of such complications and can complement contrast radiographic studies (Hamilton‐Bennett and Behr 2019).

An extensive study by Larson (2020) on the spinal morphometry of Persian cats did not report any significant interspecies differences, while another study by Klećkowska‐Nawrot et al. (2010) showed significant differences in the osseous and articular morphology of the atlas and axis vertebrae between newborn Persian cats and other feline breeds. Moreover, a study by Huizing et al. (2017) on the occipital bones and first cervical vertebra of Persian cats reported a close relationship between some cranial abnormalities, such as hydrocephalus, and the anatomy of the foramen magnum and occipital‐atlas joint in these cats. Also, another study by Okada (2009) evaluated the signs of cervical vertebral osteosarcoma in Persian cats using CT scan and MRI, reporting valuable findings. Bertolini et al. (2016) created a standard reference for the radiographic evaluation of Persian cats by conducting a comprehensive investigation of their spine using different diagnostic imaging modalities.

To the best of our knowledge, there is still no detailed and comprehensive report on the cervical vertebral anatomy of Persian cats, and the related radiographic studies are quite limited. Considering the need for detailed and specific anatomical and radiographic studies on the cervical vertebrae of Persian cats, the present study aimed to investigate the anatomical and radiological features of cervical vertebrae in Persian cats by measuring the various imaging parameters of cervical vertebrae and setting a normal range. The findings of the present study can be used to create a comprehensive reference for clinical studies and interpretation of radiographic findings in these cats

## 2 Materials and Methods

### 2.1 Ethical Consideration

This work involved the use of procedures that did not differ from established internationally recognised high standards (best practice) of veterinary clinical care for the individual animals. The study was registered under registration code# Ir.iau.urmia.rec xxxx in Ethical Committee of xxxx University, xxxx.

### 2.2 Study Design and Animals

This descriptive‐cross‐sectional study was conducted to evaluate the radiological and anatomical features of 10 adult Persian cats (5 male and 5 female) (Table 1). The Persian cats with gastrointestinal, respiratory and other infectious diseases, except head diseases, were studied. To collect samples, clinics in Tehran were requested to give Persian cats with certain mortality. Following informed consent and obtaining written approval from the owners of dead Persian cats, they were included in the study. The cats died due to disorders of the cervical area, and those younger than 12 months old were excluded from the study. The cats' age was confirmed using the dental formula by Dyce et al. (Dyce, Sack, and Wensing 2009).

**TABLE 1:** Weight and age of Persian cats.

	Male						Female					
Cats	1	2	3	4	5	mean	1	2	3	4	5	mean
Weight (Kg)	4.8	5.5	6.2	5.8	5.3	5.5	3.7	4.2	4.8	4.5	4.4	4.3
Age (month)	24	30	27	36	42	31.8	21	26	40	35	30	30.4

### 2.3 Radiographic Investigations

All cats underwent radiography in Dorsoventral (DV), Ventrodorsal (VD), left lateral and right lateral recumbency using a digital X‐ray radiography device (GXR‐SD 152 DDR, Varian N.V. Co.,Republic of Korea) with the focus‐film distance of 100 cm, peak voltage of 52 Kv, mAs of 4, and an SCI flat‐panel detector with the size of 30 × 43 cm. Moreover, Varian and Drgem software was used for image processing and tissue structure measurements.

### 2.4 Anatomical Evaluations

Following radiography, the skin and muscles of the cervical area were incised using a scalpel, and the cervical vertebrae were removed. Then, the vertebrae were rinsed with water and put in a 10% KOH solution for 5 days. Finally, the bones were bleached with H_2_O_2_ and were left to dry in sunlight for a week (Vistro et al. 2015). Each vertebra was then examined morphologically; the morphometric indices were measured using a calliper, and their mean values were recorded as follows:

‐ Vertebral Height (VH(: The distance between the floor of the vertebra and the distal end of the spinous process.
‐ Vertebral Body Height (VBH): The distance between the bottom of the vertebra and the floor of the vertebral canal.
‐ Vertebral Body Length (VBL): The distance between the lengths of the vertebral body from the cranial articular surface to the caudal articular surface.
‐ Vertebral Body Width (VBW): The width of the vertebral body at its thickest part.
‐ Spinous Process Height (SPH): The distance between the base and the apex of the spinous process.
‐ Transverse Process Width (TPW): The distance between the ends of the right and left transverse processes.
‐ Vertical Diameter of Cranial Vertebral Foramen (VDCrVF): The inner diameter of the cranial vertebral foramen (vertically) from the floor of the foramen to the ventral surface of the highest part of the vertebral arch.
‐ Transverse Diameter of Cranial Vertebral Foramen (TDCrVF): The inner diameter of the cranial vertebral foramen (horizontally) at the widest part of the vertebral arch.
‐ Vertical Diameter of Caudal Vertebral Foramen (VDCaVF): The inner diameter of the caudal vertebral foramen (vertically) from the floor of the foramen to the ventral surface of the highest part of the vertebral arch.
‐ Transverse Diameter of Caudal Vertebral Foramen (TDCaVF): The inner diameter of the caudal vertebral foramen (horizontally) at the widest part of the vertebral arch.
‐ Denticular Process Length (DPL): The length of the dental process of the axis vertebra in transverse view.

### 2.5 Statistical Analysis

The confidence interval (CI) index was used to calculate the normal ranges of the parameters of cervical bones and joints in adult Persian cats. Moreover, the parametric data were analysed using an independent t‐test and the SPSS software version 21. All variables were expressed as mean and standard deviation (SD). Also, the significance level was set at 0.05.

## 3 Results

### 3.1 Radiographic Investigation Results

**FIGURE 1:**
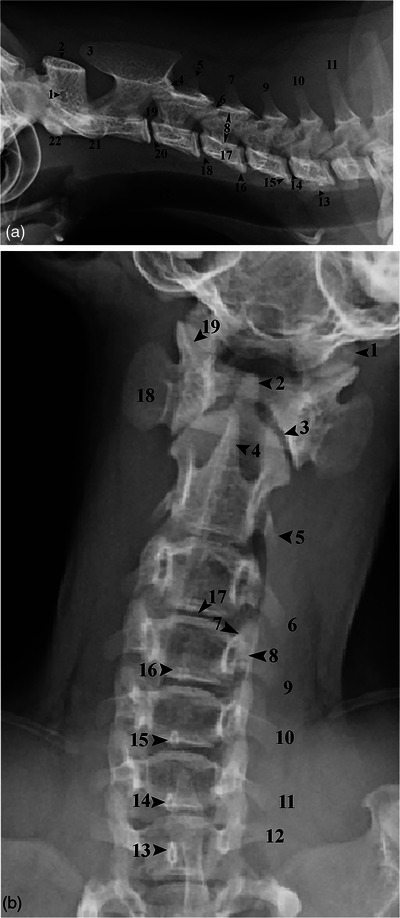
Lateral (A) and dorsoventral (B) radiographs of the neck of a 15‐month‐old male Persian cat. C_1_: Atlas vertebra, C_2_: Axis vertebra, C_3_‐C_7_: Third to seventh cervical vertebrae, T_1_: First thoracic vertebra.

According to our findings, the first cervical vertebra, the atlas (C1), of Persian cats consisted of a central arch and two broad, horizontal transverse processes. The C1 had a fully developed vertebral arch and convex articular processes in its cranial and caudal parts while lacking distinct endplates and spinous processes. Moreover, the cranial part is articulated with the occipital condyles, forming the atlanto‐occipital joint, while its caudal part is articulated with the second cervical vertebra, forming the atlantoaxial joint. Also, the transverse processes or wings were pretty broad, and each had a lateral foramen visible on radiographs. The second cervical vertebra, the axis (C2), had a long body and a large, thin and long spinous process lying on the atlantic arch. The odontoid or dens process was a long, round protrusion on the cranioventral part of the C2 extending to the ventral part of the C1. Moreover, the axis had small transverse processes, and its cranial articular processes articulated with the fovea of the atlas and atlantic caudal endplate. Also, the transverse processes of C2 were extended caudally and had a transverse foramen. The C3, C4 and C5 had bifurcated transverse processes, which resembled a broad plate in C4 and C5. However, the C6 transverse processes were broader than C4 and C5 and extended caudally. Moreover, each had small cranial tubercles. Also, the C7 had a relatively short body. The spinous processes of C4 and its caudal vertebrae were more distinct compared to the cranial vertebra. Furthermore, the notches in the caudal part of a vertebra and the cranial part of the next one formed the intervertebral foramina, which were prominent in the radiographs. However, no accessory process was observed on the foramina (Figure 1).

Having the opacity of soft tissue in radiographs, the intervertebral discs were visible between the bodies of C2 and C3 and all caudal vertebrae until the last one. However, no intervertebral disc was observed between the C1 and C2. Also, the radiographs taken from Persian cats showed the widest intervertebral space between the C4 and C5, while the narrowest was between the C2 and C3.

### 3.2 Anatomical Results (Morphology and Morphometry)

A total of seven cervical vertebrae, which were typically short, were observed in Persian cats.

#### 3.2.1 The Atlas (C1)

**FIGURE 2:**
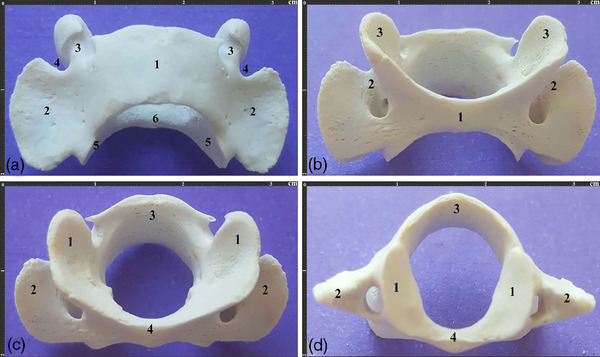
Atlas vertebra. (a). Dorsal view of the atlas vertebra of a 3‐year‐old male Persian cat. 1. Dorsal arch 2. Wing or transverse process 3. Lateral foramen 4. Wing notch 5. Caudal articular surface 6. Fovea dentis. (b). Ventral view of the same vertebra. 1. Ventral arch 2. Atlas fossa 3. Cranial articular fossa. (c). Cranial view of the same vertebra. 1. Cranial articular cavity 2. Wing 3. Dorsal arch 4. Ventral arch. (d). Caudal view of the same vertebra. 1. Caudal articular surface 2. Wing 3. Dorsal arch 4. Ventral arch.

As the widest cervical vertebra, the atlas had not‐so‐deep cranial articular cavities. Moreover, the fovea dentis was depressed and small, the dorsal arch had a lateral vertebral foramen and the alar notch was pretty small. The transverse processes or wings were extended caudally, and their caudal articular surfaces were oval, elongated and convex. Also, the ventral surfaces of the transverse processes were depressed. The C1 had no transverse foramina. Furthermore, a small dorsal tubercle was observed in the dorsal arch, while a slight and indistinct tubercle was observed in the ventral arch (Figure 2).

#### 3.2.2 The Axis (C2)

The axis had a prominent, plate‐like, one‐part spinous process, which extended cranially to the vertebral body and did not connect the caudal articular processes. Moreover, it had two caudal articular facets with their extensions lying on the caudal edge of the vertebral arch. Also, the C2 transverse process was one‐part with a transverse foramen on its base.

**FIGURE 3:**
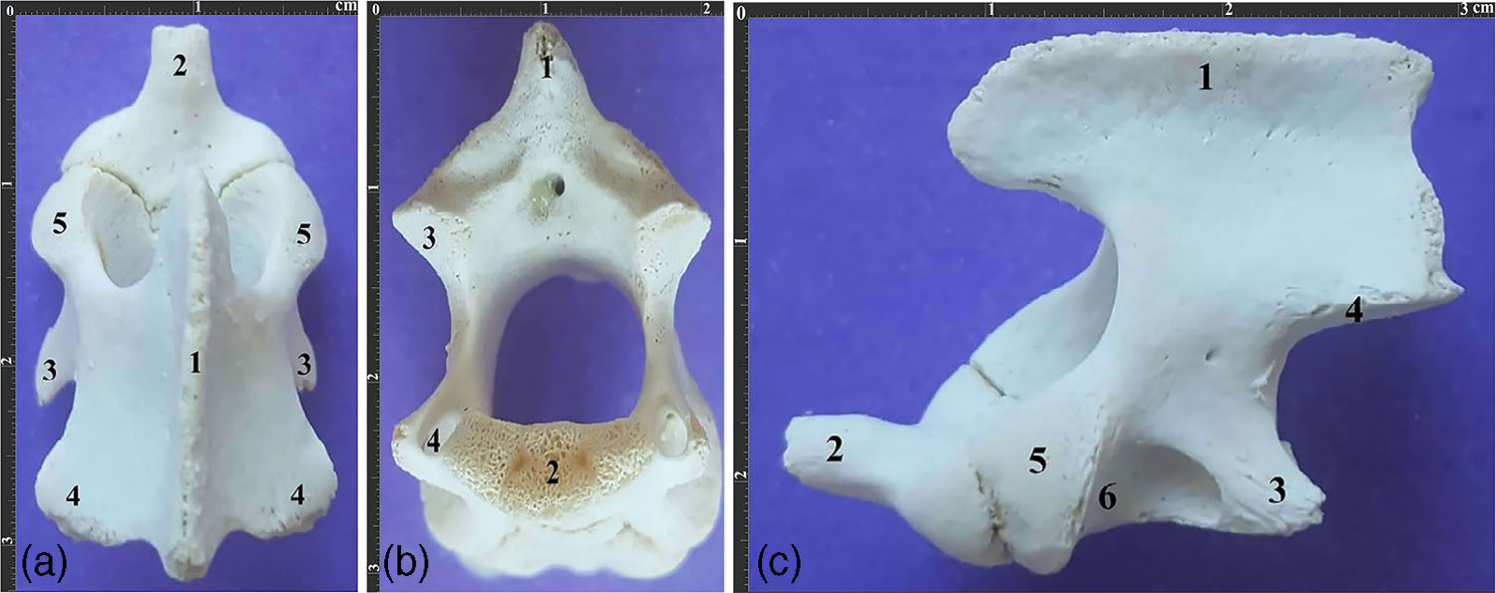
(a). Dorsal view of the axis vertebra in a 2.5‐year‐old female Persian cat. 1. Spinous process 2. Dental process 3. Transverse process 4. Caudal articular process 5. Cranial articular process. (b). Caudal view of the same vertebra. 1. Spinous process 2. Caudal articular surface 3. Caudal articular process 4. Transverse foramen. (c). Lateral view of the same vertebra. 1. Spinous process 2. Dental process 3. Transverse process 4. Caudal articular process 5. Cranial articular process 6. Vertebral body.

The transverse processes were extended caudally, while the caudal part of the vertebral body was quite large, providing a considerable surface articulating the next vertebra. The cranial articular processes were located dorsocranially to the dens process, while the cranial articular facets were oval and did not reach each other under the dens process. Moreover, no lateral vertebral foramen was observed, while the ventral surface had a tubercle instead of a crest (Figure 3). Also, the axis had the highest VBL and lowest VBW among all cervical vertebrae.

#### 3.2.3 C3, C4 and C5 (Typical Cervical Vertebrae)

These vertebrae had a low VBL with a spinous process extending cranially and growing higher in SPH from the C3 to the C5. Moreover, the transverse processes had two parts, cranial and caudal, while the caudal part was bigger and had a transverse foramen. Also, the articular facets of the cranial and caudal articular processes were one‐part, with the cranial articular facet extended dorsally while the caudal articular facet extended ventrally.

**FIGURE 4:**
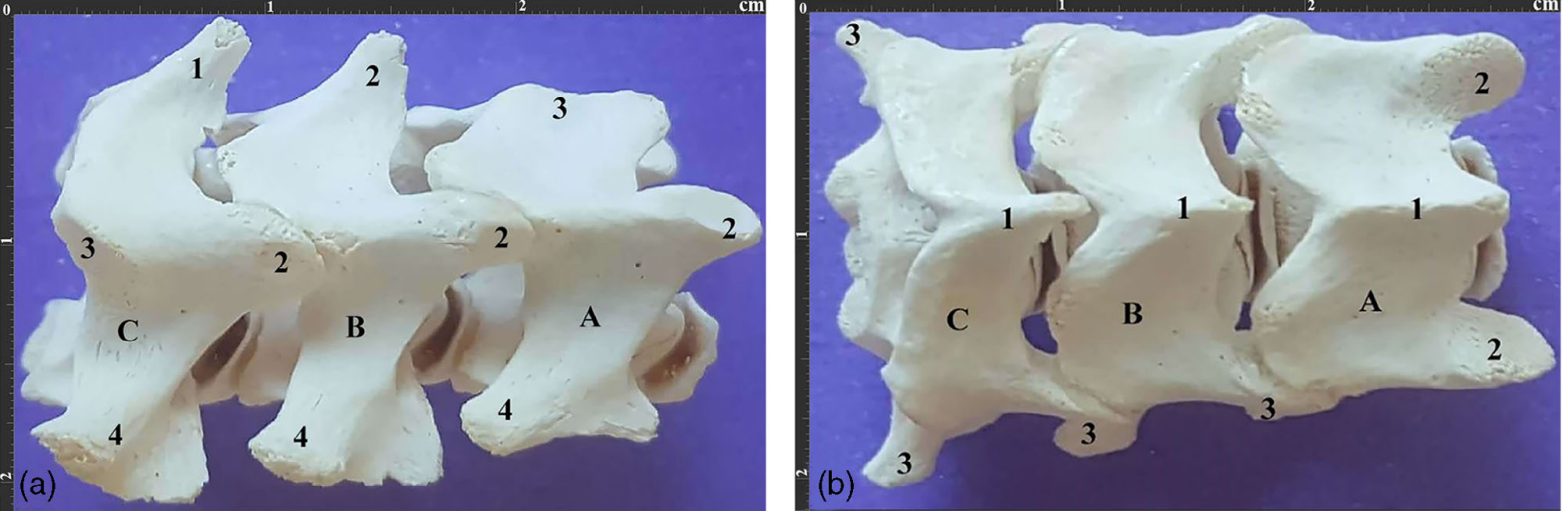
(a). Lateral view of typical cervical vertebrae of a 3‐year‐old male Persian cat. (a) 3^rd^ typical vertebra, (b) 4^th^ typical vertebra, (c) 5^th^ typical vertebra. 1. Spinous process 2. Cranial articular process 3. Caudal articular process 4. Transverse process. (b). Dorsal view of the same vertebrae. 1. Spinous process 2. Cranial articular process 3. Transverse process.

All three vertebrae had a crest‐like tubercle with moderate protuberance on their ventral surfaces. Moreover, the third typical vertebra (C5) had the lowest VBL, while the first typical vertebra (C3) had the lowest VBH and SPH among all cervical vertebrae (Figure 4).

#### 3.2.4 The Sixth Cervical Vertebra (C6)

**FIGURE 5:**
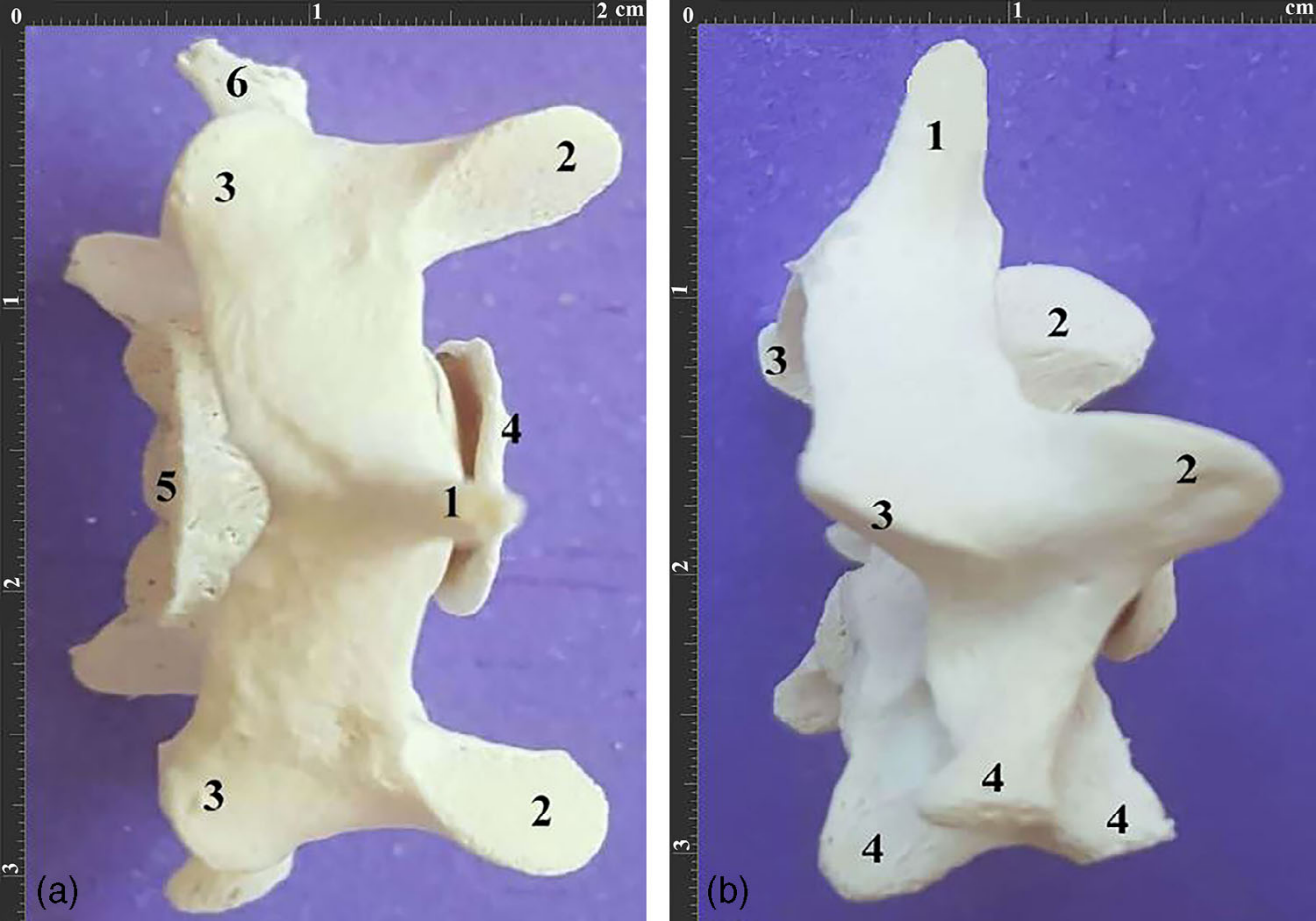
(a). Dorsal view of the 6^th^ cervical vertebrae of a 2.5‐year‐old female Persian cat. 1. Spinous process 2. Cranial articular process 3. Caudal articular process 4. Cranial articular surface 5. Caudal articular surface 6. Transverse process. (b). Lateral view of the same vertebra. 1. Spinous process 2. Cranial articular process 3. Caudal articular process 4. Three‐part transverse process.

The C6 had a higher SPH compared to other typical vertebrae, and its spinous process extended cranially, while its transverse processes consisted of three parts, with one part located dorsally and two parts located ventrocranially or ventrocaudally. Moreover, the transverse foramen connected all three parts, such as in equines and leopards. Also, this vertebra had two‐part articular facets in its cranial and caudal articular processes, which was similar to the typical vertebrae (Figure 5). A small crest‐like tuberosity was observed on the ventral surface of the C6.

#### 3.2.5 The Seventh Cervical Vertebra (C7)

**FIGURE 6:**
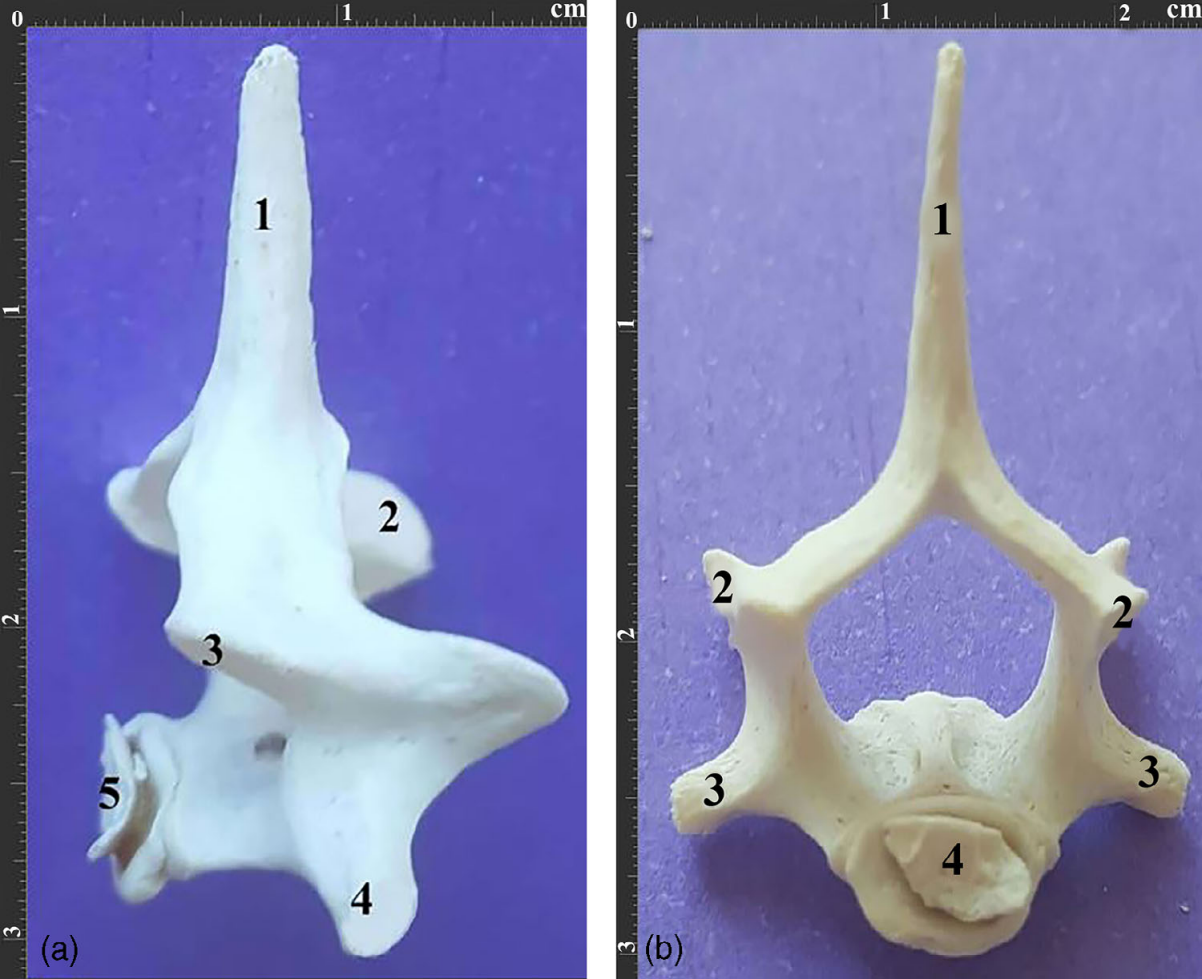
(a). Lateral view of the 7^th^ cervical vertebra of a 4‐year‐old male Persian cat. 1. Spinous process 2. Cranial articular process 3. Caudal articular process 4. Transverse process 5. Caudal articular surface. (b). Caudal view of the same vertebra. 1. Spinous process 2. Caudal articular process 3. Transverse process 4. Caudal articular surface.

The C7 had much higher SPH than the C6, and its spinous process extended cranially. The transverse processes extended quite caudally and lacked a transverse foramen. Moreover, the articular facets of the cranial and caudal articular processes were two‐part, with the cranial articular facet located dorsally and the caudal one located ventrally. Also, the caudal part of C7 had two small costal facets to articulate with the first ribs, while its ventral surface had a small crest‐like tuberosity (Figure 6). C7 had the highest VBH and SPH among all cervical vertebrae (Tables 2 and 3) (Box plots 1 and 2).

**TABLE 2:** Measurement values of cervical vertebrae parameters in male Persian cat (mm).

	Cervical vertebrae							
Parameter	C1	C2	C3	C4	C5	C6	C7	Total
VH	13.59 ± 0.51	16.39 ± 0.67	12.23 ± 0.36	15.15 ± 0.48	16.31 ± 0.45	17.82 ± 0.45	23.24 ± 0.45	16.39 ± 3.36
CrHVB	2.11 ± 0.22	3.16 ± 0.35	4.14 ± 0.41	4.22 ± 0.38	5.06 ± 0.35	4.34 ± 0.45	3.76 ± 0.35	3.82 ± 0.95
CaHVB	2.08 ± 0.30	3.74 ± 0.37	3.88 ± 0.39	3.09 ± 0.32	3.96 ± 0.33	3.75 ± 0.34	4.31 ± 0.37	3.54 ± 0.76
VBL	0.00 ± 0.00	16.93 ± 0.41	8.86 ± 0.36	8.32 ± 0.33	7.64 ± 0.24	7.66 ± 0.35	8.04 ± 0.30	8.20 ± 4.62
CrWVB	0.00 ± 0.00	13.66 ± 0.28	7.25 ± 0.33	6.90 ± 0.31	5.62 ± 0.21	6.26 ± 0.31	7.45 ± 0.29	6.73 ± 3.75
CaWVB	0.00 ± 0.00	9.05 ± 0.26	8.84 ± 0.23	8.29 ± 0.34	7.64 ± 0.24	7.48 ± 0.26	7.24 ± 0.19	6.93 ± 2.95
TPW	29.39 ± 0.67	13.32 ± 0.41	16.11 ± 0.52	17.99 ± 0.65	19.26 ± 0.53	19.42 ± 0.66	19.41 ± 0.46	19.27 ± 4.71
SPH	3.25 ± 0.19	3.25 ± 0.19	2.21 ± 0.11	4.03 ± 0.13	4.72 ± 0.15	6.81 ± 0.19	12.62 ± 0.21	5.58 ± 3.26
VDCrVF	9.09 ± 0.25	7.19 ± 0.24	5.82 ± 0.23	5.49 ± 0.21	5.46 ± 0.22	6.37 ± 0.24	6.13 ± 0.21	6.50 ± 1.22
TDCrVF	9.81 ± 0.25	6.76 ± 0.25	7.16 ± 0.23	7.44 ± 0.23	8.27 ± 0.35	7.77 ± 0.27	8.93 ± 0.24	8.02 ± 1.03
VDCaVF	9.08 ± 0.30	6.26 ± 0.29	5.36 ± 0.17	5.62 ± 0.20	6.31 ± 0.24	7.20 ± 0.25	6.84 ± 0.29	6.66 ± 1.18
TDCaVF	6.64 ± 0.25	6.79 ± 0.27	7.04 ± 0.30	7.60 ± 0.29	8.25 ± 0.28	8.77 ± 0.29	9.09 ± 0.30	7.74 ± 0.95
DPL	0.00 ± 0.00	4.44 ± 0.14	0.00 ± 0.00	0.00 ± 0.00	0.00 ± 0.00	0.00 ± 0.00	0.00 ± 0.00	0.63 ± 1.57

*Note*: The significance level was set at 0.05.

Abbreviations: (DPL) denticular process length, (SPH) spinous process height, (TDCaVF) transverse diameter of caudal vertebral foramen, (TDCrVF) transverse diameter of cranial vertebral foramen, (TPW) transverse process width, (VBH) vertebral body height, (VBL) vertebral body length, (VBW) vertebral body width, (VDCaVF) vertical diameter of caudal vertebral foramen, (VDCrVF) vertical diameter of cranial vertebral foramen, and (VH) vertebral height.

**TABLE 3:** Measurement values of cervical vertebrae parameters in female Persian cat (mm).

	Cervical vertebrae							
Parameter	C1	C2	C3	C4	C5	C6	C7	Total
VH	13.27 ± 0.53	16.11 ± 0.57	11.96 ± 0.35	14.87 ± 0.48	16.05 ± 0.45	17.59 ± 0.45	23.07 ± 0.49	16.13 ± 3.40
CrHVB	2.00 ± 0.17	2.91 ± 0.31	3.96 ± 0.39	4.01 ± 0.39	4.84 ± 0.27	4.12 ± 0.41	3.50 ± 0.40	3.62 ± 0.92
CaHVB	1.92 ± 0.26	3.57 ± 0.34	3.68 ± 0.34	2.92 ± 0.30	3.84 ± 0.24	3.62 ± 0.31	4.03 ± 0.31	3.36 ± 0.73
VBL	0.00 ± 0.00	16.81 ± 0.41	8.58 ± 0.34	8.11 ± 0.37	7.45 ± 0.34	7.54 ± 0.30	7.88 ± 0.30	8.05 ± 4.58
CrWVB	0.00 ± 0.00	13.49 ± 0.33	7.07 ± 0.26	6.58 ± 0.39	5.51 ± 0.33	6.14 ± 0.25	7.31 ± 0.33	6.58 ± 3.71
CaWVB	0.00 ± 0.00	8.95 ± 0.26	8.59 ± 0.24	8.25 ± 0.28	7.53 ± 0.18	7.41 ± 0.20	7.13 ± 0.23	6.83 ± 2.90
TPW	29.22 ± 0.61	13.17 ± 0.41	15.92 ± 0.46	17.88 ± 0.44	18.92 ± 0.66	19.06 ± 0.54	19.06 ± 0.43	19.03 ± 4.70
SPH	3.04 ± 0.17	5.21 ± 0.23	2.11 ± 0.17	3.94 ± 0.23	4.47 ± 0.15	6.45 ± 0.40	12.46 ± 0.27	5.38 ± 3.22
VDCrVF	8.95 ± 0.20	7.05 ± 0.21	5.73 ± 0.19	5.37 ± 0.19	5.39 ± 0.23	6.15 ± 0.25	6.00 ± 0.31	6.37 ± 1.21
TDCrVF	9.69 ± 0.30	6.59 ± 0.19	6.97 ± 0.22	7.13 ± 0.23	8.01 ± 0.27	7.57 ± 0.30	8.75 ± 0.30	7.81 ± 1.05
VDCaVF	8.92 ± 0.31	6.13 ± 0.26	6.21 ± 0.25	6.51 ± 0.30	6.11 ± 0.30	7.05 ± 0.31	6.58 ± 0.29	6.78 ± 0.97
TDCaVF	9.52 ± 0.37	6.58 ± 0.26	6.81 ± 0.28	7.32 ± 0.34	8.07 ± 0.29	8.60 ± 0.28	8.83 ± 0.26	7.96 ± 1.06
DPL	0.00 ± 0.00	0.00 ± 0.00	4.27 ± 0.19	0.00 ± 0.00	0.00 ± 0.00	0.00 ± 0.00	0.00 ± 0.00	0.62 ± 1.53

*Note*: The significance level was set at 0.05.

Abbreviations: (DPL) denticular process length, (SPH) spinous process height, (TDCaVF) transverse diameter of caudal vertebral foramen, (TDCrVF) transverse diameter of cranial vertebral foramen, (TPW) transverse process width, (VBH) vertebral body height, (VBL) vertebral body length, (VBW) vertebral body width, (VDCaVF) vertical diameter of caudal vertebral foramen, (VDCrVF) vertical diameter of cranial vertebral foramen, and (VH) vertebral height.

**BOX PLOT 1:**
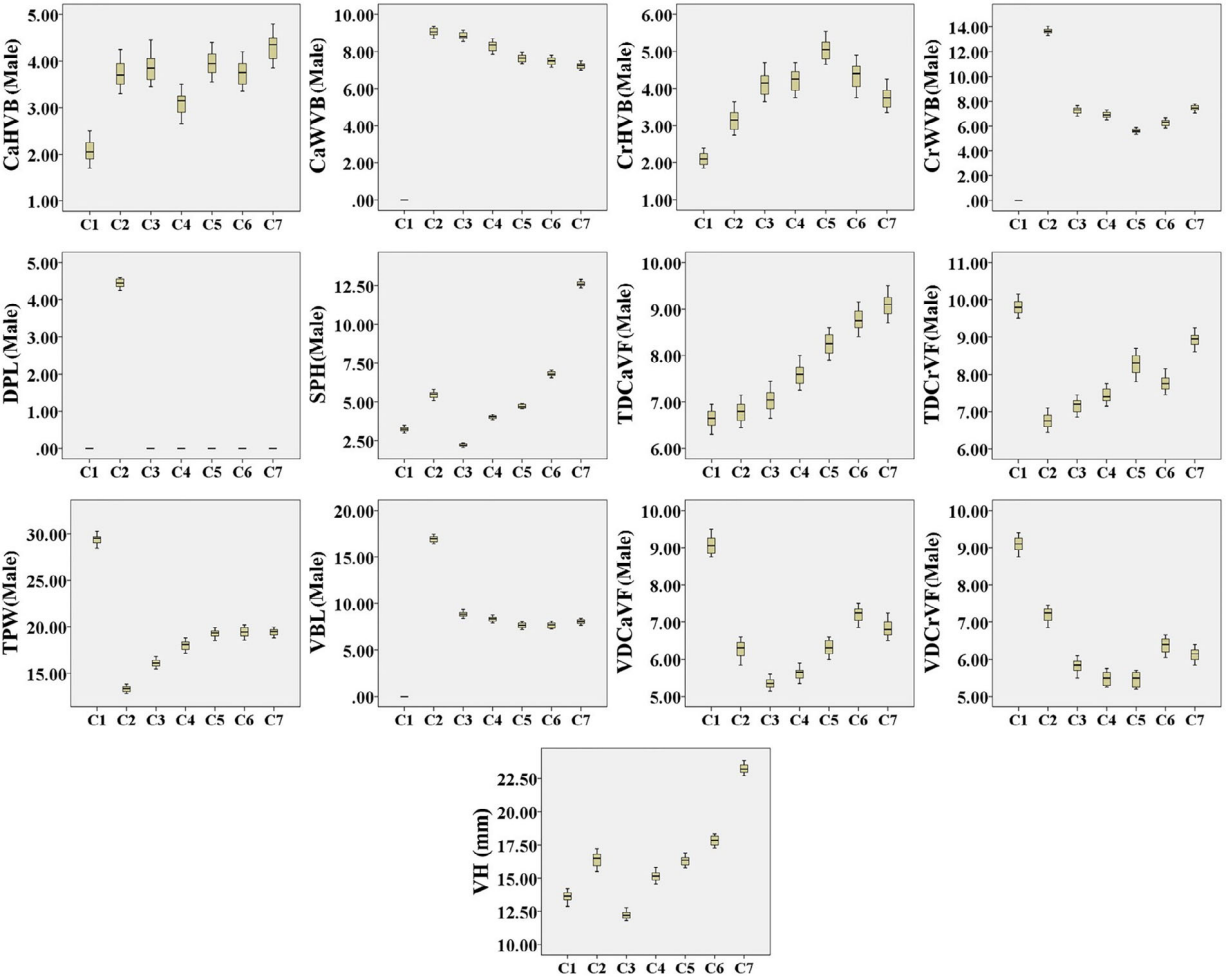
Comparison of measured parameters of cervical vertebrae in male Persian cats.

**BOX PLOT 2:**
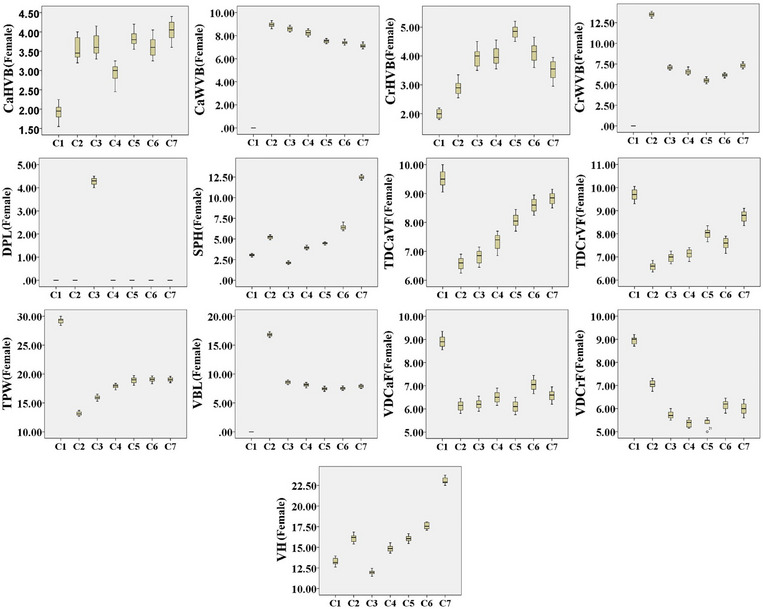
Comparison of measured parameters of cervical vertebrae in female Persian cats.

## 4 Discussion

The present study investigated the radiographic and anatomical features of the cervical vertebrae in adult Persian cats. Moreover, different parts of these vertebrae were named, described and underwent morphometrical measurements and statistical analysis.

According to our results, the C3 and C7 had the lowest and highest SPH among all cervical vertebrae, respectively, while no significant difference was observed among other vertebrae (*p* ≤ 0.05). These findings were compatible with a study by Klećkowska‐Nawrot et al. (2010). Moreover, the atlas had the highest TPW, which was significantly different from other cervical vertebrae (*p* ≤ 0.05). However, other vertebrae were not significantly different in TPW (*p* > 0.05), which was not compatible with the study by Boonsri et al. (2020). These findings indicate potential interspecies differences in cats.

On the other hand, the atlas had the highest TDCrVF and VDCrVF. However, these parameters were not significantly different among the cervical vertebrae (*p* > 0.05), which was compatible with the study by Boonsri et al. (2021). Moreover, a study by Thrall and Robertson (2022) on native cats reported that the atlas vertebra was composed of an annulus bone with two large lateral masses connected by dorsal and ventral arches, with the dorsal arch being wider than the ventral arch. Also, the dorsal arch had a small, wide and one‐part tubercle in the middle, while the ventral arch had a small tubercle in its caudal part.

However, our findings showed that in Persian cats, both dorsal and ventral tubercles were located in the caudal part of the arches, and the atlantic transverse processes extended laterally from the connection of the two arches. Moreover, these processes had foramina on their ventral surfaces, where blood vessels passed through. Also, we found transverse foramina on the caudal edge of their ventral surfaces, which was not compatible with the study by Longo et al. (2015) on the cervical vertebrae of native cats, reporting that the atlantic transverse foramen almost vertically passed through the dorsal surface of the wing. Thus, our findings on the atlas of Persian cats suggest the comparative anatomy of Persian cats with other cat breeds. These differences can be due to relatively different locations of arteries, veins and nerves passing through the atlas in different cat breeds.

The cranial and caudal ends of the atlantic transverse processes included the cranial (alar) and caudal notches, and there was a groove in the middle of the alar notch that led to the lateral foramen. This finding is reported for the first time, indicating the comparative anatomy of Persian cats with other cat breeds. Also, the atlantic lateral foramen was located in the craniodorsal part of the dorsal arch, which was not a complete foramen.

The cranial articular facet of the atlas had two cotyloid cavities articulated with the occipital condyles to allow free flexion and extension of the head, while the caudal articular facet had two shallow glenoid fossae, forming a mobile joint with the C2. The caudal part of the dorsal surface of the ventral arch had a concave fossa articulated with the axial dens process. These findings were compatible with the studies by Minto et al. (2022); Espadas, Maddox, and DeVicente (2018); and Scott, Marti, and Witte (2022), who reported no significant difference between Persian cats and other cat breeds in these anatomical features.

According to our findings, the axis had a smooth body, both ventrally and dorsally, that was compressed vertically and had a relatively high VBL. Moreover, its dens process almost touched the occipital bone, which was similar to other cat breeds and even big felines, such as leopards and tigers.

A study by Bali et al. (2009) reported that native cats had a ventral crest on their axial body, which was prominent, both cranially and caudally, and divided the ventral surface of the axis into two parts. However, the present study reported that the ventral crest on the axial body was prominent caudally, while its cranial part was indistinct. Thus, Persian cats are different from other cat breeds in the anatomy of the axial ventral crest. Moreover, we reported a thin spinous process on the cranial side, which became thick on the caudal side. Also, the cranial and caudal ends of the axis extended toward the atlas and C3, respectively, while the caudal end of the axial spinous process had a prominent tubercle with lateral protuberances extending caudally and connecting to caudal articular processes, which was compatible with previous studies.

However, a study by Indu et al. (2013) reported that the mentioned tubercle was sharp in leopards, being a different anatomical index in felines. According to their findings, the axial transverse processes were caudoventral, thin and septum‐like with a sharp ventral edge and a relatively large transverse foramen on their bases. Moreover, there were two convex, distinct and triangular cranial articular facets on the vertebral body next to the dens process. All these anatomical features were exactly reported in the axis of Persian cats.

According to our results, the VBL gradually decreased from the C2 to the C5. Moreover, the cranial articular facets of the typical vertebra were relatively convex, while their caudal articular facets were slightly concave. All these findings were compatible with the studies on other felines and even ruminants and equines. Also, the ventral crest on the bodies of the C3, C4 and C5 of Persian cats was somewhat distinct, especially at the caudal end, while it was indistinct in C6 and C7, which was similar to other cat breeds. However, it is worth noting that the ventral crest on the body of the typical vertebrae and C6 was not distinct in leopards and other felines (Indus, 2013).

On the other hand, the C3 to C7 had a cranially extended spinous process with growing SPH from the cranial vertebrae to caudal vertebrae. These findings were compatible with previous studies. However, the spinous process of Persian cats was a wide and small tubercle in C3, while it was extended almost vertically in C7. Moreover, the transverse processes of the typical cervical vertebrae were like a small plate with a cranial ventral tubercle and a caudal dorsal tubercle, which was also observed in other cat breeds. However, the C6 transverse process had a wide cranioventral tubercle, forming a sagittal plane with cranial and caudal parts separated by a notch. This unique feature of the C6 in Persian cats differentiates this breed from other cat breeds.

Also, the C7 had two small costal facets to articulate with the first ribs, while its transverse processes were single. Moreover, the C3 to C6 had distinct transverse foramina, while the C7 lacked such foramina. Also, the C7 had deep cranial and caudal vertebral notches, forming a large intervertebral foramen with the T1. These findings were compatible with the previous studies (Larson 2020; Thrall and Robertson 2022). Furthermore, the dorsal part of the articular processes of the C7 had a tubercle connected to the multifidus muscle, which was not reported in previous studies. Thus, it may be specific to Persian cats, and it is recommended to investigate this finding in future studies. Radiographic studies of Persian cat's cervical vertebrae can provide us with valuable information used in identifying its anatomical features, investigating various species of Persian cats, and evaluating their cervical pathologies. The findings of this study can be used in the diagnosis of fractures, luxation, cracks and the investigation of the healing process of fractures, as well as the evaluation of infectious and congenital diseases of the cervical vertebrae, such as arthritis and dysplasia.

## 5 Conclusion

The present study conducted a precise investigation of the cervical vertebrae of Persian cats by measuring the different parts of cervical vertebrae and joints and setting their normal ranges. The accurate and comprehensive standard ranges obtained from the present study can be used for interpretation of imaging results, clinical decision‐making and finding the normal and abnormal sizes of the cervical vertebrae and their processes in Persian cats.

## Author Contributions

**Peghah Derakhshi**: Conceptualization; Investigation; Visualization; Project administration; Resources. **Siamak Alizadeh**: Conceptualization; Investigation; Funding acquisition; Writing ‐ original draft; Writing ‐ review & editing; Visualization; Validation; Methodology; Software; Formal analysis; Project administration; Resources; Data curation; Supervision. **Mohammadreza Hosseinchi**: Data curation; Supervision; Formal analysis; Validation; Visualization; Writing ‐ original draft.

## Ethics Statement

The procedures were carried out based on the guidelines of the Ethics Committee of the International Association for the Study of Pain. This work involved the use of procedures that did not differ from established internationally recognised high standards (‘best practice’) of veterinary clinical care for the individual animals. The study was registered under registration code# (IR.IAU.URMIA.REC.1402.053) in Ethical Committee of Islamic Azad University, Urmia Branch, Iran.

## Conflicts of Interest

The authors declare no conflicts of interest.

### Peer Review

The peer review history for this article is available at https://publons.com/publon/10.1002/vms3.70109.

## Data Availability

The data that support the findings of this study are available from the corresponding author upon reasonable request.
